# Integrative Bioinformatics and Machine Learning Analysis Identifies Novel Molecular Biomarkers in Prostate Adenocarcinoma

**DOI:** 10.3390/ijms27156635

**Published:** 2026-07-25

**Authors:** Hasan Anıl Kurt, Sabire Kılıçarslan, Meliha Merve Çiçekliyurt, Serhat Kılıçarslan

**Affiliations:** 1 Department of Urology, Faculty of Medicine, Çanakkale Onsekiz Mart University, 17020 Çanakkale, Turkey; 2 Department of Medical System Biology, Graduate School of Sciences, Çanakkale Onsekiz Mart University, 17020 Çanakkale, Turkey; 3 Department of Medical Biology, Faculty of Medicine, Çanakkale Onsekiz Mart University, 17020 Çanakkale, Turkey; 4 Department of Software Engineering, Faculty of Engineering, Bandirma Onyedi Eylül University, 10200 Balıkesir, Turkey

**Keywords:** prostate adenocarcinoma, bioinformatics, machine learning, biomarker discovery, hybrid GBM+RF

## Abstract

Prostate adenocarcinoma is characterized by substantial inter-patient heterogeneity, limiting the clinical reliability of conventional diagnostic tools, including prostate-specific antigen testing. This limitation underscores the need for robust molecular biomarkers that may complement conventional diagnostic tools, highlighting the urgent need for biomarkers capable of enhancing diagnostic accuracy and enabling more precise risk stratification. In the present study, transcriptomic data from The Cancer Genome Atlas (TCGA) were analyzed using an integrative bioinformatics and machine learning pipeline., The proposed workflow was designed as a stepwise and reproducible biomarker prioritization framework in which differential expression analysis, functional enrichment, protein–protein interaction (PPI) based network interpretation, graph-convolutional feature selection, and hybrid ensemble machine learning were sequentially integrated. Differential gene expression analysis was combined with pathway enrichment (Gene Ontology (GO), Kyoto Encyclopedia of Genes and Genomes (KEGG), and Reactome), protein–protein interaction network construction, and graph-convolutional feature selection. Multiple machine learning algorithms, including Random Forest, Gradient Boosting Machine, Support Vector Classifier, Artificial Neural Network, and AdaBoost, were systematically evaluated. A hybrid ensemble model integrating Gradient Boosting Machine and Random Forest (GBM+RF) was subsequently developed. Model performance was assessed using accuracy, sensitivity, specificity, and area under the Receiver Operating Characteristic (ROC) and externally validated using the independent GSE14206 dataset. The analysis revealed a coordinated molecular pattern characterized by dysregulated cell cycle activity and enhanced interferon-mediated immune signaling. Protein–protein interaction analysis identified *STAT1* and *PLK1* as highly connected network hub genes within immune-related and cell-cycle-associated modules. Among the evaluated models, the hybrid GBM+RF framework achieved the highest predictive performance on the TCGA dataset, with AUC: 0.9526; Accuracy: 97.49%. External validation using the GSE14206 dataset confirmed the robustness of this model (AUC: 0.9156; Accuracy: 91.53%). These findings support a broader multi-gene candidate signature in prostate adenocarcinoma, in which machine learning prioritized genes such as *XAF1*, *APP*, *RPA3*, *IFIH1*, *UBE2D2*, *RSAD2*, *KIF2C*, and *PLK1*, while *STAT1* and *PLK1* provided complementary network-level biological relevance. The proposed framework provides a robust and transferable strategy for biomarker discovery and precision oncology.

## 1. Introduction

Urological malignancies, especially prostate cancer, constitute a substantial burden on the male cancer burden worldwide [1]. Urothelial carcinomas make up an overwhelming majority, about 90%, of these cases. The high rate of relapse of these tumors has made extended clinical surveillance indispensable. Though prostate cancer remains the most common solid tumor in men and its diagnosis is the leading cause of cancer death among them, it is also one of the most important factors in cancer mortality in general [2]. Although most cases are typically slow and gradual, it is becoming increasingly clear that some subtypes. PSA-based screening is beneficial; the debate remains open, although it is widely used, concerns persist regarding overdiagnosis and the resulting overtreatment [3,4].

Early diagnosis plays an important role in the clinical management of prostate cancer by supporting timely risk assessment and treatment planning. Conventional diagnostic tools such as PSA testing, digital rectal examination, imaging-based evaluation, and biopsy remain central to clinical practice [5,6]. However, these tools may sometimes provide ambiguous or non-specific findings, particularly in biologically heterogeneous cases. Therefore, molecular biomarkers derived from transcriptomic data may serve as complementary tools to support clinicians in early diagnosis, risk stratification, and individualized decision-making. One example is the PSA test, which does not have enough specificity for making a final clinical decision and often results in overdiagnosis or unnecessary treatments. Likewise, standard cytology is often unable to identify low-grade cancers; it poses a continuous challenge to doctors who meet such slow-growing but still troublesome cases. The absence of these diagnostic modalities highlights the great importance of implementing more refined, molecular-based methods.

Currently, diagnostic approaches largely rely on conventional markers, which have not been conclusively demonstrated to serve as reliable predictors of the disease. Therefore, the integration of high-dimensional transcriptomic data represents a significant paradigm shift, with the potential to improve diagnostic accuracy and reduce ambiguity in clinical outcomes [7]. Besides intratumoral cellular diversity, within a single tumor, and the usually slow development of these cancers, their very nature of being biologically unpredictable makes the challenge of obtaining a diagnosis that is both timely and accurate even more difficult. Although imaging-based and clinical diagnostic approaches provide essential information for prostate cancer assessment, molecular-level data may further improve the biological interpretation of clinically ambiguous cases. In this context, transcriptomic biomarkers may complement existing diagnostic tools rather than replace them.

It is therefore natural that the convergence of the complexities of living beings and the constraints of technology should give rise to molecularly guided diagnostic systems. One very effective way to exceed the limits of traditional morphology, based on imaging, is to integrate transcriptomic data with artificial intelligence, thereby enabling a profound understanding of tumor behavior from the very beginning.

The problems encountered are not only the result of technology but also a manifestation of the clinical complexity that comes with the heterogeneity of prostate tumors. Tumor variability is prevalent in patients and sometimes even in the tumor itself. The cancer’s development is frequently slow, and at times very slow, so traditional imaging approaches are insufficient; consequently, there is growing awareness that prostate adenocarcinoma is a molecularly heterogeneous tumor in terms of transcripts and molecules, which cannot be inferred by clinical criteria. Large-scale gene expression datasets from the Gene Expression Omnibus (GEO) and The Cancer Genome Atlas (TCGA) are widely utilized in bioinformatics analyses to identify novel biomarkers and signal networks involved in tumor development. Researchers have begun integrating transcriptomic data with pathway enrichment and network-based analyses, highlighting the relevance of these analyses to their biological studies of mechanisms driving carcinogenesis. Therefore, the use of differential expression analysis along with Gene Ontology (GO), Kyoto Encyclopedia of Genes and Genomes (KEGG), and Reactome pathway enrichment leads to the discovery of candidate genes and functional modules related to the aggressiveness of the disease.

In addition, the merging of bioinformatics data with predictive modeling through machine learning not only creates a strong data-driven structure connecting molecular biology to computational oncology but also improves diagnostic findings and survival classification. The standard approaches are still PSA, DRE, TRUS, and cystoscopy [8]. In clinical diagnostics, inherent biological heterogeneity often results in ambiguous or borderline findings. This ambiguity poses a substantial challenge even for highly experienced clinicians, especially when they must decide on a definitive treatment plan. Moreover, the issue becomes more complicated as one of the hallmark features of the classical diagnostic procedures is very high levels of invasiveness or extremely expensive, which significantly hampers their implementation in routine clinical practice.

To address such challenges, machine learning (ML) frameworks have been developed as sophisticated tools for deciphering the intricate, non-linear relationships concealed in the complex biomedical data of high dimensions. These computer-based systems are highly efficient in processing the massive amount of “big data,” demonstrating two main capabilities: firstly, they enhance the diagnostic precision, and, secondly, and perhaps even more importantly, they provide useful prognostic information about the likely evolution of the patients. By shifting from merely identifying to forecasting, these methods pave the path for more individualized and less costly patient care [9,10].

This research opens the molecular landscape of prostate adenocarcinoma in several ways. One is a high-resolution transcriptomic profiling of the tumor samples, and the other is biology that inspired computational analysis. Firstly, we used gene expression data from TCGA to elucidate the layer of genetic dysregulation that progressively increases with disease advancement. By focusing our attention on genes according to their patterns of differential expression, we aimed to pinpoint the transcriptomic signatures that specifically drive cancer development from the stage of localized disease to that of advanced adenocarcinoma. This multi-integrative approach ensures that the markers thus obtained are biologically relevant and are not simply the result of random noise in the data. Next, functional enrichment and protein–protein interaction analyses were conducted to identify biological elements and molecular networks associated with the differentially expressed genes.

Overall, the results suggested that transcriptomic changes did not stem from a single cause but rather demonstrated a coordinated adjustment across multiple signaling pathways linked to cell cycle regulation, mitotic progression, p53 signaling, and interferon-driven immune responses—a comprehensive analytical suite. Then, machine learning-based prioritization was introduced to determine if the biologically filtered genes were still truly informative across various computational setups. This step showed that a subset of candidate genes was repeatedly selected across multiple algorithms, indicating their relative stability within the analyzed transcriptomic feature domain. Validation with an independent cohort (GSE14206) showed the robustness of the gene signature identified, as it kept on delivering diagnostic efficacy at a level of accuracy of almost 90%. Such external validation also proves the strength of the panel under different experimental conditions.

Ultimately, this investigation highlights biological coherence and multi-methodological consensus as opposed to narrow, model-specific fine-tuning. Prioritizing features that are invariant across different computational architectures, we offer a practical and highly transferable framework for biomarker discovery. Thus, the molecular drivers that we have identified here are not just a result of some kind of statistical noise but rather are stable, clinically relevant targets that point to further research in prostate adenocarcinoma.

The novelty of the present study does not lie in generating a new public dataset, but rather in constructing a structured, reproducible, and biologically grounded analytical pipeline for prostate adenocarcinoma biomarker prioritization. In contrast to conventional approaches in which differential expression analysis, pathway enrichment, PPI network analysis, and machine learning models are often evaluated as independent components, the proposed framework integrates these analytical layers into a sequential workflow. First, statistically significant DEGs were identified from the TCGA-PRAD dataset. Their biological relevance was then evaluated using GO, KEGG, Reactome, and PPI network analyses. Subsequently, graph-convolutional feature selection was applied to prioritize genes within a network-aware context rather than evaluating them as isolated molecular features. Finally, multiple machine learning models, including the Hybrid GBM+RF ensemble, were used to assess the discriminative stability of the resulting gene signature, followed by external validation using GSE14206.

### 1.1. Motivation

Prostate adenocarcinoma is a molecularly complex and heterogeneous disease, rendering its biological behavior and progression difficult to predict. Although the prostate-specific antigen test is widely used in clinical practice, it is insufficient to capture the molecular complexity of prostate cancer. PSA levels do not directly reflect tumor-specific transcriptomic alterations, dysregulated cell cycle activity, interferon-mediated immune signaling, DNA damage response, androgen receptor-related transcriptional changes, or tumor microenvironment-related molecular heterogeneity. Therefore, transcriptomic biomarkers may provide complementary molecular information that can support early diagnostic assessment, risk stratification, and individualized clinical decision-making in prostate adenocarcinoma [11].

Although large-scale transcriptomic studies have generated extensive RNA-level data on prostate adenocarcinoma, most existing studies predominantly employ either descriptive interpretation or Predictive machine learning independently. Integrating these complementary strategies is essential for achieving a more complete understanding of the disease. The present study seeks to bridge this gap by establishing an integrated framework that combines the identification of differentially expressed genes, the biological interpretation of their functions and interactions, and computational gene prioritization. Rather than relying solely on statistical filtering, the functional roles and interaction patterns of the candidate genes were examined, and machine learning was subsequently used to assess their discriminative relevance. To the best of our knowledge, this is one of the first studies to apply a well-organized, stepwise model for prostate adenocarcinoma in which conventional bioinformatics analyses guide and constrain the subsequent machine learning-based gene prioritization, rather than their independent execution. In this way, the study aims to identify molecular markers that are both biologically interpretable and computationally robust, providing a strong rationale for future translational and biomarker-focused research in prostate cancer.

### 1.2. Literature Review

Limitations of conventional diagnostic approaches in prostate cancer have accelerated the shift toward more precise and clinically meaningful biomarkers driven by transcriptomic data and machine learning methodologies. Recent prostate cancer studies show a growing shift toward data-driven biomarker discovery strategies that integrate transcriptomic data with machine learning. In particular, the combination of spatial transcriptomics, pseudotime analysis, and machine learning has enabled the identification of reliable urinary biomarkers in large patient cohorts. These approaches have achieved high predictive accuracy (AUC = 0.92) and have been further validated in independent cohorts [12]. This aligns with emerging standards emphasizing external validation and translational applicability.

Complementing these findings, Prostate Cancer Diagnosis Using Machine Learning Classifiers Based on Non-invasive Urinary RNA Biomarkers (PCASSO) proposes a non-invasive diagnostic paradigm based on urinary RNA biomarkers, thereby addressing the well-documented limitations of prostate-specific antigen (PSA) testing, including low specificity and high false-positive rates [13]. By benchmarking six machine learning classifiers, the study reinforces the viability of AI-assisted diagnostics without the need for invasive procedures, including digital rectal examination.

At the prognostic level, integrative transcriptomic analyses have gained considerable traction. Integration of Single-Cell and Bulk RNA-Sequencing Data Reveals the Prognostic Potential of Epithelial Gene Markers for Prostate Cancer demonstrates how combining single-cell and bulk RNA sequencing across multiple datasets enables the derivation of an epithelial gene signature with strong predictive value for biochemical recurrence-free survival [14]. Similarly, multi-omics frameworks integrating diverse analytical pipelines, such as DESeq2, WGCNA, and consensus machine learning models, have identified immune-related and prognostic signatures with high reproducibility.

At the mechanistic level, studies such as Integrative Analysis of Molecular Mechanisms in Prostate Cancer via Single-Cell RNA Sequencing and WGCNA provide valuable insights into tumor microenvironment dynamics, highlighting key genes (e.g., *CNPY2*, *CPE*, *DPP4*, and *IDH1*) as potential therapeutic targets [15]. These findings are further supported by investigations into intra-tumoral heterogeneity, where machine learning-assisted Cox regression models have been used to construct prognostic signatures linked to treatment resistance and disease progression [16].

From a data infrastructural perspective, large-scale integration efforts such as the development of a Longitudinal Prostate Cancer Transcriptomic and Clinical Data Linkage demonstrate the feasibility of linking transcriptomic profiles with electronic health records and insurance data across nearly 93,000 patients, facilitating longitudinal analyses and enhancing clinical decision-making pipelines [17]. These developments reflect a broader convergence toward reproducible computational workflows in oncology research.

Importantly, the transition from traditional clinical markers such as PSA and Gleason score toward molecular and AI-driven biomarkers is strongly supported by systematic reviews, which emphasize the growing relevance of coding and non-coding RNA species (mRNA, microRNA, and lncRNA) in precision oncology [18]. This paradigm shift is also driven by clinical concerns regarding overdiagnosis and overtreatment, where novel biomarkers such as *PCA3* and *TMPRSS2*-*ERG*, alongside active surveillance strategies, are increasingly recognized [19,20].

Overall, the convergence of multi-omics data integration, machine learning, and large-scale clinical datasets is redefining prostate cancer diagnostics and prognostics, enabling more precise, non-invasive, and clinically actionable biomarker development. In conclusion, integrating transcriptomic technologies with machine learning has enabled the development of more accurate diagnostic and prognostic biomarkers for prostate cancer. Non-invasive approaches and multi-omics integration hold considerable promise for overcoming the limitations of PSA-based methods; however, further large-scale validation remains essential prior to clinical implementation.

## 2. Results

**Figure 1:**
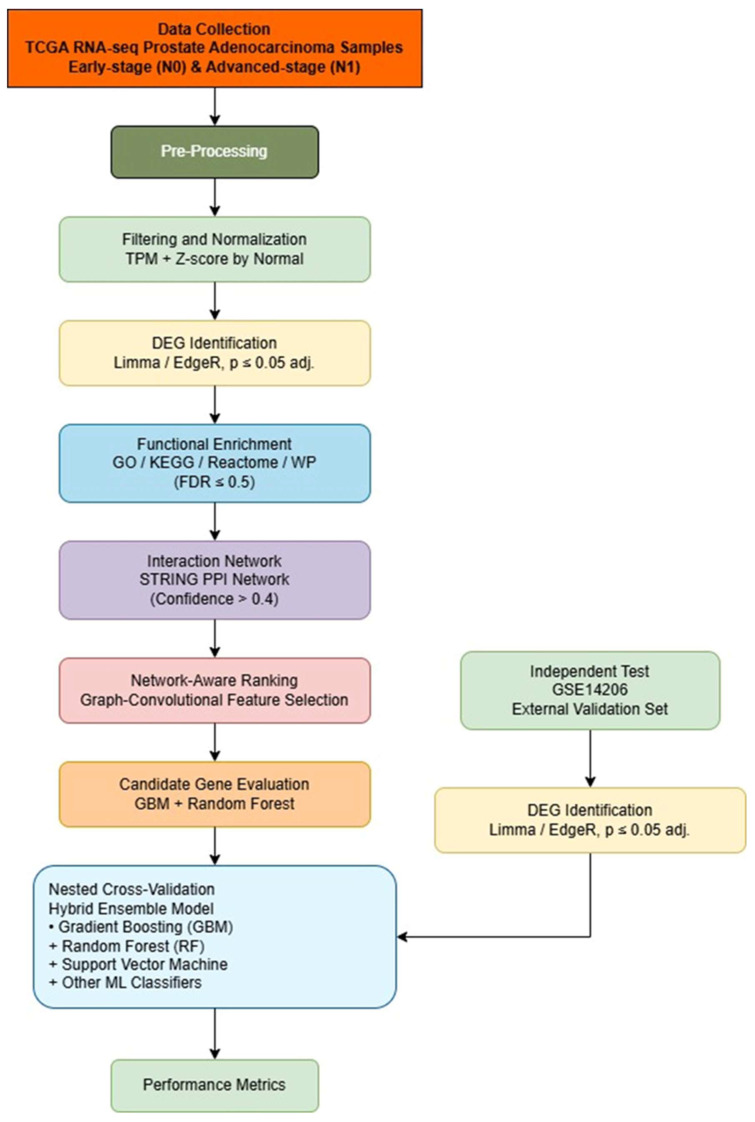
Flow chart of the bioinformatics and machine learning methodology used.

This study used an integrative bioinformatics and machine learning framework to identify transcriptomic alterations associated with prostate adenocarcinoma progression. RNA-seq data from the TCGA-PRAD cohort were analyzed to identify DEGs between early- and advanced-stage tumors using predefined Log_2_FC and Benjamini–Hochberg adjusted *p*-value thresholds. Significant genes were subjected to functional enrichment, PPI network analysis, graph-convolutional feature selection, and machine learning-based prioritization. The diagnostic performance of the selected biomarker signature was evaluated utilizing multiple classifiers and externally validated in the independent GSE14206 cohort. The overall workflow is presented in Figure 1.

As summarized in Figure 1, the analytical workflow consisted of two main stages. In the first stage, Z-score-normalized RNA-seq expression data from the TCGA-PRAD cohort were analyzed to identify transcriptomic alterations associated with prostate adenocarcinoma progression. Node-negative (N0) and node-positive (N1) samples were compared to detect differentially expressed genes associated with nodal status.

**Figure 2:**
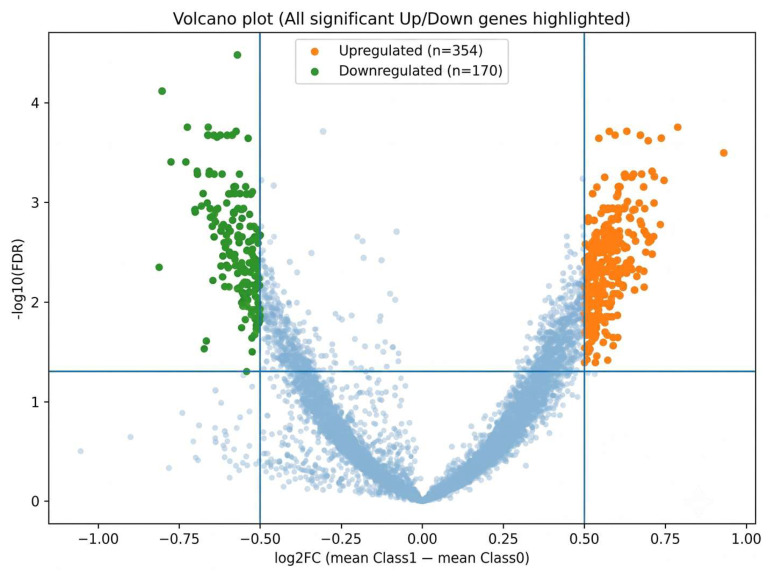
Among the candidate genes, 170 were downregulated and 354 were upregulated.

The TCGA prostate adenocarcinoma dataset was analyzed to identify candidate genes. The preprocessed Z-score-normalized expression matrix included 20,512 genes; 1,974 genes were excluded, and was stratified according to nodal status, comprising 342 nodes-negative (N0) and 78 nodes-positive (N1) prostate adenocarcinoma samples. DEG analysis showed that, in total, 524 genes were differentially expressed: 354 were upregulated and 170 were downregulated (Figure 2). Machine learning-based analysis was applied only to DEGs to prioritize biologically relevant candidates.

The identified DEGs were then used for downstream bioinformatics analyses. Functional enrichment and PPI network analyses were performed to characterize the biological processes, pathways, and interaction patterns associated with these genes.

In the second stage, machine learning models were applied to the previously identified DEGs. This step was used to support gene prioritization and to evaluate the discriminative relevance of the selected genes. Before model training, graph-convolutional feature selection was applied to rank DEGs by incorporating network-derived information.

Several machine learning algorithms were evaluated, including Decision Tree, Random Forest, Support Vector Classifier, Artificial Neural Network, Gradient Boosting, and AdaBoost. A cross-validation framework was used to assess model performance and reduce the effect of random data splitting.

Feature rankings from different models were compared to identify genes recurrently prioritized across algorithms. Hybrid ensemble methods were also evaluated, and the GBM+RF model achieved the highest overall performance. The selected gene signature was further assessed using the independent GSE14206 validation dataset.

Overall, this workflow combined DEG analysis, bioinformatics interpretation, graph-based feature selection, and machine learning-based prioritization to identify candidate genes with both computational relevance and biological support.

### 2.1. Bioinformatics Analysis

KEGG and Reactome Pathway analyses were performed to identify major oncogenic pathways associated with the selected candidate genes. This dual-database approach provided complementary insights into regulatory pathways involved in prostate adenocarcinoma pathogenesis. Furthermore, the functional properties of the identified genes were systematically analyzed using Gene Ontology categories, and those involved in Biological Process, Cellular Component, and Molecular Function were evaluated.

The connectivity and functional interactions among candidate genes and their encoded proteins were further investigated using the STRING database. Protein–protein interaction (PPI) networks were constructed to characterize the physical and functional relationships involved in cellular signaling. Centrality analysis based on degree was used to detect key subnetworks and central hub proteins. PPI networks were visualized by Cytoscape 3.9.x. The major pathways identified through these analyses are summarized in Table 1, Table 2 and Table 3.

Table 1 indicates that the enrichment profile was mainly associated with antiviral defense responses, innate immune signaling, cell-cycle regulation, and p-53-related proliferative control. Interferon-stimulated genes (*ISGs*), including *DDX58*, *IFIH1*, *STAT1/STAT2*, *MX1/MX2*, *OAS1/OAS3*, *IRF7*, and *IFNG*, were among the pathway-associated genes, suggesting the presence of an interferon-driven transcriptional landscape in prostate adenocarcinoma.

In addition to this immune-related profile, a number of genes were identified as key regulators, such as *CCNE2*, *PLK1*, *CDC20*, *CDKN2A* and *CASP3,* involved in cell-cycle regulation, mitotic progression, and DNA damage response. Mitosis-related pathways, including oocyte meiosis-related terms, were enriched, suggesting dysregulation of chromosome segregation and genome stability mechanisms. Furthermore, the enrichment of pathways related to adaptive immune signaling, such as the *IL12B*, *CD80* and *IFNG* pathways, annotated under Type I diabetes mellitus, indicates adaptive immune signaling as another potential contributor to the local molecular environment.

Overall, the enrichment results indicate a mixed molecular profile characterized by interferon-mediated immune signaling and perturbation of the cell-cycle/p53-related pathways in prostate adenocarcinoma.

Table 2 indicates that the enriched pathways were predominantly associated with mitotic cell-cycle regulation, M-phase progression, kinetochore function, spindle assembly checkpoint activity, and chromosomal segregation. Pathway analysis revealed several genes, such as *PLK1*, *CDC20*, *CDCA8*, *CENPA/CENPE*, *BUB1*, *KIF2C*, and *PTTG1*, that were represented in pathways related to sister chromatid separation and transition from prometaphase to anaphase. These findings indicate a prominent enrichment of mitotic progression-related transcriptomic features in prostate adenocarcinoma.

Enrichment was also observed for pathways involved in *APC/C*-mediated degradation of cell-cycle proteins, including *APC/C–CDC20*-related regulatory processes. Several genes identified in these pathways, such as *WEE1*, *CDC25C*, *PKMYT1*, and *CCNE2*, are known to participate in G2/M phase transition and cell-cycle checkpoint regulation. Additionally, RHO GTPase effector pathways were enriched, indicating the involvement of cytoskeleton-related and cell division-associated signaling processes in the observed transcriptomic profile.

**Figure 3:**
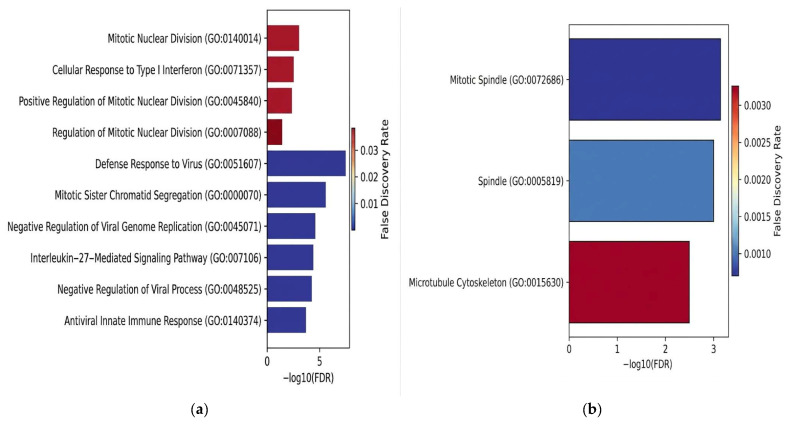
(**a**) GO biological process and (**b**) GO cellular component.

Overall, Table 2 supports a cell-cycle-dominant enrichment pattern characterized by mitotic progression, chromosomal segregation, checkpoint regulation, and *APC/C–CDC20*-related cell-cycle control. Figure 3 presents the GO enrichment analysis of the differentially expressed genes.

The GO Biological Process enrichments analysis revealed enrichment of both cell division-related processes and interferon-associated responses. Two signatures were identified as significantly enriched: mitotic nuclear division and sister chromatid segregation, which reflect proliferation and an innate antiviral response, respectively, suggesting the presence of immune-related and proliferative transcriptional programs in prostate adenocarcinoma (Figure 3a). GO Cellular Component analysis revealed that the genes found were mainly related to the mitotic spindle, microtubule cytoskeleton and other structures related to cell division. These results reflect the enrichment of cellular categories in the framework of the “mitotic apparatus” and “microtubule associated” (Figure 3b).

The PPI network constructed from differentially expressed genes is shown. Nodes represent proteins/genes, and edges indicate known or predicted interactions based on experimental evidence, curated databases, and literature sources. Network analysis revealed two prominent functional modules. The upper cluster comprises genes primarily associated with cell cycle regulation and mitotic progression (*PLK1*, *CDC20*, *BUB1*, *KIF2C*, *KIF20A*, *RACGAP1*, *UBE2C*, *RRM2*, and *DLGAP5*). The lower cluster consists of genes mainly involved in interferon signaling and immune response pathways (*STAT1*, *IFNG*, *CXCL10*, *MX1*, *RSAD2*, *XAF1*, and *FCGR3A*). Node colors indicate distinct functional modules, and node size reflects relative connectivity (degree centrality) within the network. These findings suggest that dysregulation of both cell cycle progression and interferon-mediated immune responses is a key biological process in the studied condition.

**Figure 4:**
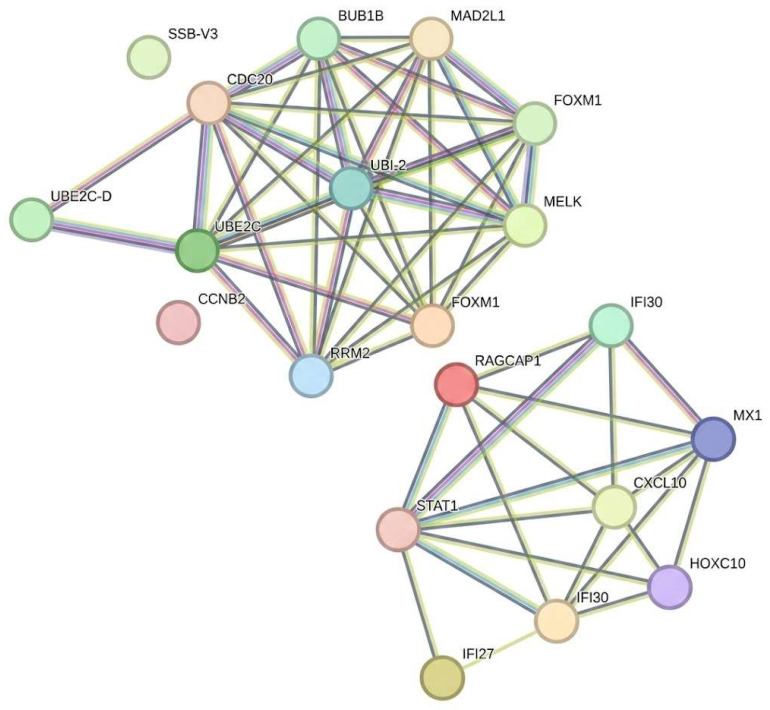
Protein–protein interaction (PPI) network of differentially expressed genes and functional modules.

The protein–protein interaction (PPI) network of the differentially expressed genes is shown in Figure 4. The network demonstrated a high degree of functional connectivity, with a clustering coefficient of 0.82 and significant PPI enrichement *p*-value, indicating The network demonstrated a high degree of functional connectivity, with a clustering coefficient of 0.82 and a significant PPI enrichment *p*-value, indicating that the candidate genes were more interconnected than expected by chance.

Network analysis revealed two major functional modules. The first module included genes mainly associated with cell-cycle regulation and mitotic progression, such as *PLK1, CDC20*, *BUB1*, *KIF2C*, *KIF20A*, *RACGAP1*, *UBE2C*, *RRM2*, and *DLGAP5*. The second module included genes primarily related to interferon signaling and immune response pathways, including *STAT1*, *IFNG*, *CXCL10*, *MX1*, *RSAD2*, *XAF1*, and *FCGR3A*. Node size reflected degree centrality within the network, and *PLK1* and *STAT1* appeared as highly connected hub genes within their respective functional modules.

Overall, the PPI network supported the presence of two major transcriptomic modules involving mitotic cell-cycle regulation and interferon-mediated immune signaling. The comprehensive network structure of the differentially expressed genes is shown in Figure 4.

### 2.2. Machine Learning Analysis

Machine learning analysis was used as a complementary step to evaluate the previously identified DEGs. The aim was to assess the discriminative ability of these candidate genes and to identify ones prioritized across methods. Graph-based feature selection was applied before model training. According to their relative importance, the ranked genes generate a reduced set of candidate genes for further analysis.

Several classification algorithms were then evaluated, including Decision Tree, Random Forest, Support Vector Classifier, Artificial Neural Network, Gradient Boosting, and AdaBoost. Model performance was assessed using cross-validation. Accuracy, sensitivity, specificity, and the AUC were used as performance metrics. The comparative results are summarised in Table 3.

The outputs of the different algorithms are compared to identify genes that were consistently selected across models. Genes that were repeatedly prioritized by multiple classifiers were considered to be more stable candidate features. A hybrid ensemble model combining GBM and Random Forest was also evaluated. This model demonstrated the most consistent performance of all the tested approaches. Table 4 represents the top 20 genes identified by each algorithm.

Table 3 reports model performance on the TCGA dataset. AUc values ranged from 0.8421 to 0.9526. Decision Tree and Ada Boost classifiers ranked lowest, whereas Artificial Neural Network, Support Vector Machine, and Gradient Boosting Machine fell in intermediate range. Random Forest and the hybrid GBM+RF ensemble reached the upper bound of discriminative accuracy, with the latter attaining 97.49% accuracy and an AUC of 0.9526. These findings indicate that ensemble-based models outperformed single-classifier approaches on this dataset.

Table 4 lists the top-ranked genes selected by each machine learning algorithm from the 524 DEGs. The table was used to evaluate cross-model recurrence rather than to establish a universal gene ranking across all models.

**Figure 5:**
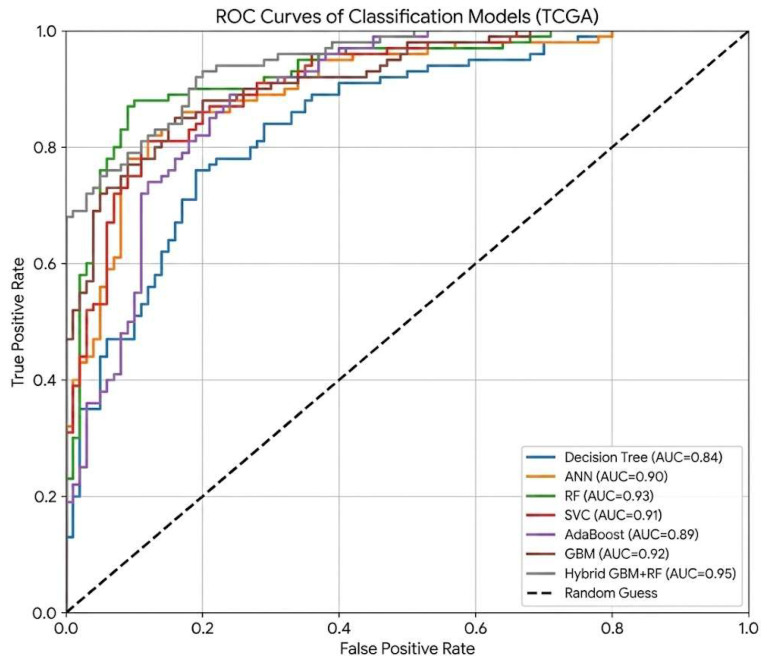
Roc Curve Performance.

Several genes, including *XAF1*, *APP*, *RPA3*, *KIF2C*, *PLK1*, *STAT1*, *RSAD2*, *IFIH1*, and *UBE2D2*, were recurrently selected by multiple algorithms, although their relative ranking differed across models. Among these genes, *PLK1* and *STAT1* were also identified within relevant modules in the PPI network. Therefore, Table 4 should be interpreted as a model-specific feature prioritization table that helps identify recurrent candidate genes across algorithms, rather than as evidence for a single universal gene ranking. The ROC curves of the evaluated models are shown in Figure 5.

As shown in Figure 5, ROC curve analysis across the eight architectures yielded AUC values ranging from 0.84 to 0.95. All models markedly exceeded random classification performance. The hybrid GBM+RF ensemble achieved the highest discriminative capacity (AUC = 0.95), followed by XGBoost (0.94), Random Forest (0.93), and standalone GBM (0.92). Single classifier approaches such as SVC, ANN, AdaBoost and Decision Tree exhibited comparatively lower predictive performance.

These findings support the use of ensemble-based strategies, which appear to optimize the sensitivity–specificity trade-off more effectively than individual algorithms. The hybrid model’s superior AUC implies robust class separation across the full range of decision thresholds, a property consistent with improved diagnostic reliability.

### 2.3. Validation on Independent Dataset (GSE14206)

The GSE14206 dataset was used as an independent external validation cohort to test the robustness of the identified gene signature. The classification accuracy varied from 86.67% for the Decision Tree to 91.53% for the Hybrid GBM+RF model. The Hybrid GBM+RF model also achieved an AUC of 0.916, indicating stable and reliable external validation performance. Re-evaluation of feature rankings in the independent GSE14206 validation cohort showed that *XAF1*, *APP*, *RPA3*, *KIF2C*, *PLK1*, *RSAD2*, *IFIH1*, and *UBE2D2* were repeatedly prioritized across models, supporting the stability of a broader multi-gene candidate signature. These findings suggest that the identified candidates are relatively stable across independent datasets and different models.

The genes listed in Table 5 and Table 6 represent model-specific features identified by each machine learning algorithm. These tables were used to evaluate cross-model recurrence and candidate gene consensus, not to imply that some genes occupied the highest ranks in all algorithms. Accordingly, genes repeatedly detected across multiple models were considered more stable consensus candidates.

## 3. Discussion

Globally, prostate cancer is still among the most common malignancies diagnosed in men. Despite significant advances in diagnostics and treatment techniques, mortality rates are still high, particularly for those with late-stage diseases. This persistent clinical burden means that new molecular targets, together with biology-driven therapeutics, are needed. Within this context, key regulatory molecules such as *STAT1* and *PLK1* have attracted attention due to their importance in control of proliferation, division, survival and the metastatic potential of cancer cells.

In this study, we applied a comprehensive computational scheme involving large-scale bioinformatics and several machine learning approaches, such as Decision Trees, Artificial Neural Networks, Random Forest, Gradient Boosting Machine, Support Vector Classifier, and AdaBoost. A wide range of modelling was used to support cross-validating candidate gene markers and limiting algorithm-specific bias. The machine learning models prioritized a broader set of recurrent candidate genes including *XAF1*, *APP*, *RPA3*, *IFIH1*, *UBE2D2*, *RSAD2*, *KIF2C*, and *PLK1*, whereas *STAT1* and *PLK1* were most clearly supported by PPI network analysis as hub genes within immune-related and cell-cycle-associated modules. Thus, *STAT1* and *PLK1* represent biologically meaningful features of prostate adenocarcinoma rather than algorithm-specific artifacts [21]. Moreover, combining Ensemble and Hybrid learning approaches helped us obtain more consistent and reliable results. By evaluating the data through different models rather than relying on a single algorithm, we were able to minimize the impact of random fluctuations and improve the overall robustness of our findings.

In addition to the statistical measures, our analysis revealed a small, coherent cluster of genes that function as a single unit rather than as individual units. These genes were involved in major cellular checkpoints, regulating cell cycle progression, chromosome segregation, and cell cycle regulation, as determined through functional enrichment and network analysis.

Within the network-level interpretation, *PLK1* and *STAT1* may represent complementary biological modules rather than the top-ranked machine learning features. *PLK1* regulates several steps of the cell cycle, including the initiation of mitosis, the spindle assembly and the segregation of the chromosomes; its deregulation is associated with cell cycle defects and a process called “mitotic catastrophe” in malignancies. In contrast, *STAT1* functions in the interferon-activated pathway, transmitting immune signals and controlling the interferon-induced expression of inflammatory and antiviral genes. This network-level co-occurrence suggests a possible relationship between cell-cycle dysregulation and interferon-mediated immune signaling during prostate cancer progression; however, this interpretation should be regarded as hypothesis-generating and not as direct evidence of a causal *STAT1–PLK1* regulatory axis.

The role of *STAT1* in cancer biology is still unclear, and there are reports of both suppressive tumor and tumor-promoting functions in various types of cancers in different contexts [22]. This variation highlights the importance of further investigating *STAT1*-dependent signaling networks and their possible relationship with cell-cycle-associated regulators such as *PLK1* in prostate cancer models [23].

Consistent with these observations, elevated nuclear *STAT1* expression has been documented in high-grade prostate cancer, castration-resistant disease, and distant metastases, where it correlates with early recurrence and prostate cancer-specific mortality. *STAT1* also mediates interferon-induced tumor-suppressive responses, highlighting the context-specific nature of its function [24]. Experimental studies reveal that prostate cancer tissue contains elevated *STAT1* expression compared to normal prostate epithelium, while *STAT1* knockdown by RNAi decreases malignant cell growth in vitro, which fits with a pro-oncogenic function in certain disease settings [25].

The disruption of *JAK/STAT* signaling in mutations has a negative effect on responses of the immune cells to interferon treatment, also against prostate cancer and other cancers, making interpretation of *STAT1* even more difficult [26]. Thus, there is a need for a thorough analysis of the various modulators of cells and environments such as PTEN deletion and defects in DNA damage response pathways to clarify its contradictory role [27,28]. The interaction between *STAT1* and *PLK1* in prostate cancer with PTEN loss can be studied to better understand the rewiring of their functions in the development of the disease and therapeutic resistance, which can help inform rational design of targeted and combination therapies.

Our data collectively indicate that the highly malignant prostate adenocarcinoma phenotypes do not emerge from individual molecular mutations but instead result from coordinated dysfunction of cell cycle mechanisms and immune-related pathways. This study brings together the fields of bioinformatics, network biology and ensemble machine learning to deliver mechanistically informed and computationally sound findings on prostate cancer molecular pathogenesis that inform future translational and therapeutic advances.

Together, our data support a model in which highly malignant prostate adenocarcinoma phenotypes arise not from isolated molecular alterations but from coordinated dysregulation of cell cycle and immune-related signaling mechanisms. By integrating bioinformatics, network biology, and ensemble machine learning, this study provides mechanistically grounded, computationally robust insight into the prostate cancer pathogenesis.

### Limitations

This study has several important limitations that should be considered when interpreting the findings. First, the analysis is entirely based on publicly available transcriptomic datasets (TCGA and GEO) and lacks experimental validation at the protein or functional level. Therefore, the biological roles of the identified candidate genes, particularly *STAT1* and *PLK1*, remain to be confirmed through in vitro and in vivo studies.

Second, the GSE14206 data set was used as an independent external validation set, but the sample size is limited. Thus, the results of this study may have limited statistical and generalizability. To establish if this gene signature is effective and clinically applicable in real world scenarios, larger multi-center cohorts with well-curated clinical information is required. Moreover, the two TCGA-PRAD and GSE14206 were produced by two different transcriptomic technologies, RNA-seq and microarrays. Some platform-dependent effects were removed by preprocessing the data in each platform, mapping probes to genes, selecting common genes, and normalizing each dataset by z-scores, but removing all platform-dependent effects is not possible. Advanced batch-effect correction methods such as ComBat or Remove Unwanted Variation (RUV) were not employed in the present study. Therefore, residual platform-specific technical variation may have influenced the external validation results, which should be interpreted as supportive rather than definitive evidence of cross-platform generalizability. Thus, the proposed signature should be revisited in larger independent cohorts using potentially more similar transcriptomic platforms in future studies.

Third, to avoid overfitting, a nested cross validation framework was used, but the combination of high dimensional transcriptomic data and machine learning models is inherently prone to over-optimization. Although efforts have been made to prevent this from happening, this possibility cannot be ruled out and must be considered in future studies with the use of prospective validation design.

Fourth, the study focuses exclusively on transcriptomic alterations and does not incorporate other layers of molecular regulation such as epigenomics, proteomics, or metabolomics. Given the complex and multi-layered nature of cancer biology, integrative multi-omics approaches may provide a more comprehensive understanding of disease mechanisms.

Finally, the clinical utility of the identified biomarkers was not directly assessed in relation to established clinical parameters such as PSA levels, Gleason score, tumor stage, or patient survival outcomes. Future studies integrating molecular findings with clinical variables are essential to determine the translational relevance and potential implementation of these biomarkers in clinical practice.

## 4. Materials and Methods

### 4.1. Material

In this study, the prostate adenocarcinoma cohort from the Cancer Genome Atlas PanCancer Atlas project (TCGA, PanCancer Atlas, 2018) was retrieved from cBioPortal for Cancer Genomics [29]. The downloaded data files were originally provided in tab-separated text format and were subsequently converted into comma-separated values format to ensure compatibility with standard downstream bioinformatics and statistical analysis tools. An independent validation cohort, GSE14206, was used to assess the reproducibility of findings. The GSE14206 dataset was generated using the GPL-570 microarray platform and contains expression measurements for 54,675 genes. For the present analysis study, a total of 50 samples were included, consisting of 15 normal prostate tissue samples and 35 prostate adenocarcinoma samples. The GSE14206 dataset was utilized as an external validation cohort to evaluate the robustness and generalizability of the proposed analytical framework and biomarker signature. Because TCGA-PRAD and GSE14206 were generated using different transcriptomic platforms, platform-specific preprocessing and a common-gene harmonization step were applied before external validation, as described in the preprocessing section.

The Cancer Genome Atlas (TCGA) represents an extensive repository of genomic data that pairs gene expression readings with clinical facts taken from patients. The expression portion stores, for every patient, a Z-score for each of 20,531 genes—the scores come from 494 prostate adenocarcinoma cases. Table 7 shows how a Z-score appears.

In Table 7, Hugo_Symbol represents the official gene symbol assigned to each transcriptomic feature. The column headers beginning with “TCGA” correspond to anonymized patient/sample identifiers from the TCGA-PRAD cohort. The numerical values represent Z-score-normalized mRNA expression levels for each gene in each sample. Positive Z-score values indicate relatively higher expression compared with the reference distribution, whereas negative Z-score values indicate relatively lower expression. Therefore, Table 1 provides a representative segment of the normalized gene expression matrix used for downstream differential expression and machine learning analyses.

The downloaded Prostate Adenocarcinoma (TCGA, PanCancer Atlas) datasets consist of 63 columns and 494 rows, respectively. In both datasets, variables with all values marked as “NA”, as well as those unrelated to the scope of the study such as patient identification (ID) and hospital codes were excluded from the analysis.

The study was conducted in two sequential stages. First, differential expression and bioinformatics analyses were performed to identify genes and pathways associated with prostate adenocarcinoma. In the second stage, machine learning–based analyses were applied exclusively to the previously identified DEGs to prioritize genes with higher discriminative relevance and to provide supportive validation.

### 4.2. Pre-Processing Process

TCGA-PRAD was used as the discovery dataset for mRNA expression analysis. RNA-Seq expression data were retrieved from cBioPortal using the RNA-Seq V2 RSEM-normalized expression pipeline. Gene expression values were analysed as Z-score normalized data, which represents the relative deviation of each gene’s expression level in a given tumor sample from the reference expression distribution provided by the dataset. Positive Z- score indicates increase gene expression whereas negative Z-score indicate decreased gene expression relative to the reference cohort. The TCGA-PRAD dataset included RNA-Seq expression profiles from 494 prostate adenocarsinoma patients and was integrated with corresponding clinical data for downstream analysis gene exhibiting increase or decrease expression patterns were identified based on Z-score normalized RNA-Seq data. For external validation, the GSE14206 dataset was analyzed independently. In this cohort, genes were considered upregulated Log_2_FC > 0.5 or downregulated Log_2_FC < 0.5 when FDR < 0.05 after Benjamini-Hochberg correction.

Because the discovery and validation cohort were generated utilizing different transcriptomic platforms, a cross-platform harmonization step was performed before external validation. TCGA-PRAD RNA-Seq data and GSE14206 microarray data were proceed independently according to their platform-specific characteristics rather than being compared directly at the raw expression level. In GSE14206, raw CEL files were preprocessed using the Robust Multi-array Average (RMA) algorithm via the affy Bioconductor package (version 1.88.0) in R, which performs background correction, log_2_ transformation, and quantile normalization at the probe level. In GSE14206, probe identifiers were mapped to official gene symbols using the GPL 570 annotation files. Multiple probes mapping to the same gene were summarized using the mean expression value, whereas probes without valid gene symbol annotations were removed. After probe to gene conversion, only overlapping genes between the TCGA-derived DEG feature set and the GSE14206 dataset were retained. To reduced platform related scale effects, both datasets were subjected to gene wise Z-score normalization prior to model testing. Principal component analysis (PCA) was performed on the combined expression matrix after Z-score normalization to verify that samples did not cluster exclusively by platform. Thus, external validation was performed in a shared common gene feature space rather than by directly comparing raw RNA-Seq microarray expression values.

Machine learning analyses were carried out as a secondary and supportive step. Only previously identified DEGs were included as input features. The aim of this analysis was not prediction or clinical classification, but prioritization of genes based on their relative contribution within the DEG set.

### 4.3. Bioinformatics Processes

Functional enrichment analysis of the identified candidate genes was performed using the Enrichr/enrichR package in RStudio 2025.x. Gene ontology analyses were conducted for Biological Process, Cellular Component, and Molecular Function categories. Enriched terms with an adjusted *p*-value ≤ 0.05 were considered statistically significant, and cancer related pathways were prioritized for further interpretation. Protein–protein interaction (PPI) analyses were carried out using the STRİNG database via network Analysis, with a high-confidence interaction score threshold of ≥0.900. The network topology was evaluated utilizing degree-based metrics to identify central hub proteins. The resulting PPI networks were visualized using Cytoscape.

### 4.4. Machine Learning Algorithms

In this study, multiple machine learning methods were used to classify prostate cancer samples based on gene expression profiles, including AdaBoost, Random Forest (RF), Gradient Boosting Machine (GBM), Support Vector Classifier (SVC), and the C5.0 decision tree (DT) [30,31,32,33,34]. These models were selected due to their ability to handle complex and high-dimensional biological data, particularly microarray datasets [35,36,37,38].

#### 4.4.1. Proposed Hybrid GBM+RF Model

In this study, a hybrid machine learning framework combining the complementary strengths of GBM and RF algorithms was developed to support gene prioritization and classification in prostate adenocarcinoma.

The proposed hybrid GBM+RF model integrates two powerful ensemble learning algorithms in a sequential manner. In the first stage, GBM captures complex, nonlinear, and interactive relationships between features and the target variable by producing sequential weak learners $h_{m}(x)$. GBM starts with a base prediction function $F_{0}(x)$ and, at each iteration, computes the negative gradient (pseudo-residual) $r_{\mathrm{im}}$ to identify the direction of steepest descent in the loss function L(y, F(x)). Each new weak learner is trained on these residuals, and the model is updated using a step size γ_m_ and learning rate ν (Equation (1)):

(1) $$F_{m}\left(x\right)=F_{m-1}\left(x\right)+v\cdot \gamma_{m}\cdot h_{m}\left(x\right)$$

At the end of this process, intermediate prediction scores ($\hat{y}\_GBM$) are generated for each observation. In the second stage, these scores are concatenated with the original feature matrix X to create an extended feature space $Z\;=\;[X,\;\hat{y}\_GBM]$. The RF model is then trained on this extended space using bootstrap sampling and random feature selection, building B independent decision trees $T_{b}\left(x\right)$. The final hybrid model prediction is (Equation (2)):

(2) $${\hat{y}}_{Hybrid}\left(x\right)=\frac{1}{B}\sum_{b=1}^{B}T_{b}\left(\left[x,{\hat{y}}_{GBM}\right]\right)$$

**Figure 6:**
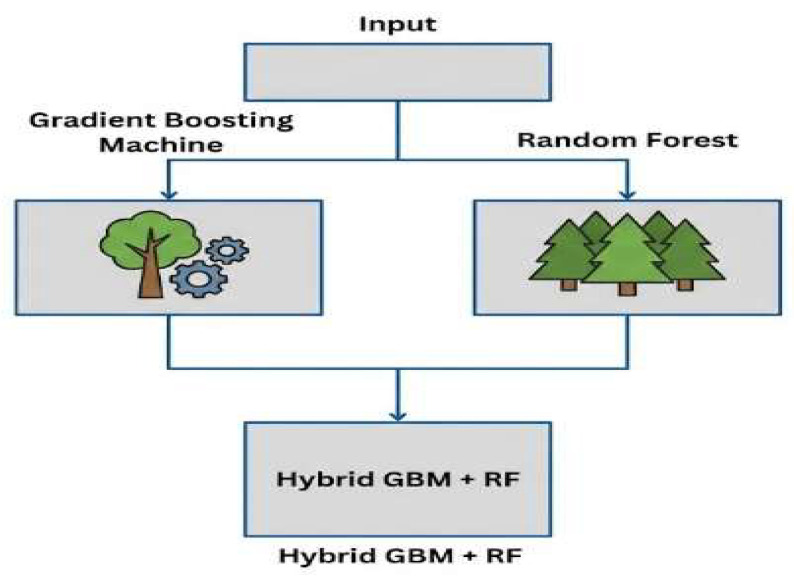
Proposed Model.

The architecture works in two steps. The gradient boosting module learns strong patterns that lower bias. The random forest module lowers variance and improves generalization. The two steps yield a model that keeps high accuracy, stays robust plus stays interpretable on biomedical data that are noisy, high-dimensional and class-imbalanced. Figure 6 shows the proposed model.

In the second phase of the study, machine learning approaches were applied to support gene prioritization and diagnostic classification rather than to predict theoretical biological activities. Following differential expression analysis, graph-convolutional feature selection was applied exclusively to the identified differentially expressed genes (DEGs), using network-derived information to guide gene ranking and selection. This step enabled the prioritization of genes based on their relative importance within the network context, without performing explicit joint modeling of gene expression and interaction data. Model stability and generalizability were confirmed by nested cross, validation. The authors tried multiple classification algorithms such as DT, RF, GBM, SVC, and Artificial Neural Network (ANN) models for the task at hand. In addition to single classifiers, hybrid ensemble methods were also explored, and the hybrid GBM+RF model achieved the most stable and strong performance based on different evaluation metrics. Model performance was measured against standard classification metrics, such as accuracy, sensitivity, specificity, F1, score, and area under the ROC curve (AUC). Ultimately, the hybrid GBM+RF framework was identified as the most dependable method for DEG, based prostate adenocarcinoma classification in this research.

Hyperparameter tuning was performed within a nested cross-validation framework to reduce model-selection bias and minimize overfitting. In the outer loop, the dataset was divided into training and test folds to estimate model performance, whereas the inner loop was used for hyperparameter optimization using only the training portion of each outer fold. The optimization criterion in the inner loop was the area under the receiver operating characteristic curve (AUC), because the primary aim of the study was to evaluate discriminative performance. A grid search method was applied to set the main hyperparameters of the GBM and RF components. The search spaces and final selected parameters for the GBM and RF components of the Hybrid GBM+RF model are summarized in Table 8.

#### 4.4.2. Graph-Convolutional Feature Selection (GCFS) for Gene Prioritization

Gene Correlation, based Feature Selection (GCFS) [39] ultimately results in a ranked list of genes as well as a reduced, top-priority gene subset that can be utilized in further analyses. Basically, the approach attributes an importance score to each gene by considering both its expression signal and its significance in the interaction network and then ranks genes accordingly. Hence, the output will be a feature set that is not only smaller but also biologically more coherent and can be used in the next machine learning steps, which, in most cases, lead to improved model stability and make the selected biomarkers a lot easier to interpret in a network context.

Before training the model, GCFS was employed to cut down the number of features and discard the genes that were not informative or redundant. The quality of the model was checked using nested cross, validation. In the inner loop, the feature selection and hyperparameter optimization were done, while the outer loop was used to get an unbiased estimate of how well the model will perform on new data. We have calculated 95% confidence intervals (CI) for the AUC values of the GCFS+RF+GBM framework based on a robust bootstrapping method to confirm the statistical soundness of our results.

Moreover, DeLongs test was utilized to compare the diagnostic efficacy of the integrated GCFS+RF+GBM architecture with that of the single classifiers formally. Such comparative statistical analysis was necessary to establish whether the proposed hybrid framework provided a significant predictive advantage over the conventional, single, model approaches.

Model architectures built around trees, in particular RF and GBM, were chosen due to their outstanding capability of operating in high, dimensional feature spaces. GBM is a type of tree, structured model that has been heavily fine, tuned for efficiency and speed, yet RF is a model that increases the overall stability and noise, and resistance of the model. Besides that, we relied on SVC for their characteristics of separating patterns in high, dimensional space with great accuracy and on the C5.0 model, which is able to provide very interpretable, human, readable rules, based logic, to further diversify our set of prediction engines.

The best result was obtained by combining RF and GBM with the help of a regression meta, classifier. Such an ensemble tactic allowed to efficiently utilize the strong sides of the two algorithms resulting in better accuracy and AUC metrics in the detection of prostate cancer signatures. The findings highlight that ensemble methods based on decision trees are extremely capable of mapping the non, linear nature of genomic datasets.

Concerning the computational environment, the entire bioinformatics preprocessing and transcriptomic data normalization were run in R (version 4.3.1) through the Bioconductor ecosystem, including the limma, edgeR, and genefilter libraries. On the other hand, the creation and fine, tuning of the machine learning models took place in Python (version 3.11), making use of the scikit, learn, GBM, and XGBoost packages.

The proposed pipeline was not designed to generate a new transcriptomic dataset, but rather to provide an integrative analytical framework that transforms publicly available transcriptomic data into a biologically interpretable and computationally prioritized biomarker signature. The key distinction from simple database querying is that each analytical layer constrains and validates the subsequent step. Differential expression analysis first narrows the initial disease-associated gene space; functional enrichment and PPI analyses then provide functional and network-level interpretation; graph-convolutional feature selection subsequently ranks genes using network-aware information; and machine learning models finally evaluate the discriminative capacity of the prioritized genes across samples. The overall framework therefore functions as a reproducible decision pipeline rather than a descriptive list of database-derived genes.

## 5. Conclusions

This study proposes a novel integrative analytical framework combining large-scale bioinformatics and machine learning strategies to elucidate transcriptomic alteration associated with prostate adenocarcinoma.

By integrating DEG, functional enrichment, network-based interpretation, and ensemble learning, genes with consistent biological relevance and clinical value were identified. The results support a broader multi-gene candidate signature, including *XAF1*, *APP*, *RPA3*, *IFIH1*, *UBE2D2*, *RSAD2*, *KIF2C*, and *PLK1*, while *STAT1* and *PLK1* were particularly supported by PPI network analysis as biologically relevant hub genes.

*PLK1* was linked to abnormal cell-cycle regulation and mitotic instability, while *STAT1* was found to be a prominent player in interferon-mediated immune signaling. Together, the machine learning-prioritized genes and the PPI-supported hub genes suggest a molecular phenotype characterized by proliferative activity, mitotic dysregulation, and immune-related transcriptomic changes The current observations indicate that there is a coordinated alteration of proliferative and immune-related pathways that can lead to aggressive prostate adenocarcinoma, not a single molecular event.

The strong predictive performance of the hybrid GBM+RF ensemble model provides additional methodoligal support for the proposed framework. In the TCGA discovery set, the model performed well with a classification accuracy of 97.45% and AUC of 0.95, while in the independent GSE14206 validation set, the classification accuracy was 91.57% and AUC was 0.92. The cross-platform validation results validate the robustness and potential generalizability of the identified gene signature.

However, pathway enrichment analysis only identified a few pathways that were statistically significant post-multiple testing correction. It is important to note that results at the pathway level must be considered as possible future research directions and not as proof of causal disease mechanisms. Moreover, the findings are based on silico analyses, which reduces the immediate clinical applicability, and lack detailed clinical information.

The broader candidate gene panel, including *XAF1*, *APP*, *RPA3*, *IFIH1*, *UBE2D2*, *RSAD2*, *KIF2C*, *PLK1*, and *STAT1*, should be further validated in clinically well-characterized cohorts using qPCR, immunohistochemistry, proteomic profiling, and functional assays. Use of molecular features in combination with known clinical markers such as PSA, Gleason score, and tumor stage will further enhance the specificity of diagnosis and accuracy of prognosis. Furthermore, the discovery of an interconnected cell cycle–immune regulatory axis may offer a translational foundation for combination therapies involving both proliferative and immune mechanisms.

In general, this study offers a biologically plausible and mathematically sound approach for biomarker discovery in prostate adenocarcinoma and suggests the potential for using transcriptomic information combined with machine learning in designing clinically relevant diagnostic and therapeutic approaches.

## Figures and Tables

**Table 1:** KEGG pathway analysis.

Term ID	Term Description	False Discovery Rate	Matching Proteins in Your Network (Labels)
hsa05162	Measles	0.0022	*[‘DDX58’, ‘STAT1’, ‘MX2’, ‘STAT2’, ‘MX1’, ‘ADAR’, ‘IFIH1’,* *‘CCNE2’, ‘OAS1’, ‘CASP3’, ‘OAS3’, ‘IL2RA’, ‘IRF7’, ‘IL12B’]*
hsa05164	Influenza A	0.0022	*[‘RSAD2’, ‘DDX58’, ‘STAT1’, ‘MX2’, ‘STAT2’, ‘MX1’, ‘ADAR’,* *‘IFIH1’, ‘CXCL10’, ‘IFNG’, ‘OAS1’, ‘CASP3’, ‘OAS3’, ‘IRF7’, ‘IL12B’, ‘KPNA2’]*
hsa04110	Cell cycle	0.0088	*[‘CDC20’, ‘CDKN2B’, ‘WEE1’, ‘PTTG1’, ‘CCNE2’, ‘ESPL1’,* *‘CDKN2A’, ‘CDC26’, ‘PLK1’, ‘CDC25C’, ‘PKMYT1’, ‘BUB1’]*
hsa05160	Hepatitis C	0.0166	*[‘RSAD2’, ‘DDX58’, ‘STAT1’, ‘MX2’, ‘STAT2’, ‘MX1’, ‘IFIT1’,* *‘CXCL10’, ‘IFNG’, ‘OAS1’, ‘CASP3’, ‘OAS3’, ‘IRF7’]*
hsa04115	p53 signaling pathway	0.0233	*[‘RRM2’, ‘CCNE2’, ‘CDKN2A’, ‘CASP3’, ‘SIAH1’, ‘CCNG1’,* *‘PTEN’, ‘IGF1’]*
hsa04114	Oocyte meiosis	0.0233	*[‘CDC20’, ‘PTTG1’, ‘CCNE2’, ‘STAG3’, ‘ESPL1’, ‘CDC26’, ‘PLK1’,* *‘IGF1’, ‘CDC25C’, ‘PKMYT1’, ‘BUB1’]*
hsa04940	Type I diabetes mellitus	0.0270	*[‘PTPRN2’, ‘IFNG’, ‘CD80’, ‘LTA’, ‘IL12B’, ‘CPE’]*

**Table 2:** Reactome pathwaysenrichment.

Term Description	False Discovery Rate	Matching Proteins in Your Network (Labels)
Separation of Sister Chromatids	1.82 × 10^−10^	*[‘UBE2C’, ‘CDCA5’, ‘PLK1’, ‘CDCA8’, ‘CENPA’, ‘PSMA7’, ‘CDC20’, ‘CENPE’, ‘DSN1’, ‘KIF18A’, ‘TUBA3C’, ‘PTTG1’, ‘ESPL1’, ‘PSMA1’, ‘TUBB3’, ‘NUP85’, ‘CENPI’, ‘CDC26’, ‘NUF2’, ‘KIF2C’, ‘BUB1’]*
M Phase	2.43 × 10^−10^	*[‘CDCA5’, ‘CDCA8’, ‘SMC4’, ‘CENPA’, ‘PSMA7’, ‘CDC20’, ‘DSN1’, ‘TUBA3C’, ‘PTTG1’, ‘NUP85’, ‘TUBB3’, ‘CDC26’, ‘CEP70’, ‘ENSA’, ‘NUF2’, ‘NEK2’, ‘BUB1’, ‘UBE2I’, ‘ODF2’, ‘UBE2C’, ‘PLK1’, ‘HAUS4’, ‘KIF23’, ‘CENPE’, ‘KIF18A’, ‘PSMA1’, ‘ESPL1’, ‘CENPI’, ‘KIF2C’, ‘KIF20A’, ‘NCAPD3’]*
Mitotic Anaphase	1.20 × 10^−11^	*[‘UBE2I’, ‘UBE2C’, ‘CDCA5’, ‘PLK1’, ‘CDCA8’, ‘CENPA’, ‘PSMA7’, ‘CDC20’, ‘CENPE’, ‘DSN1’, ‘KIF18A’, ‘TUBA3C’, ‘PSMA1’, ‘PTTG1’, ‘ESPL1’, ‘NUP85’, ‘CENPI’, ‘TUBB3’, ‘CDC26’, ‘NUF2’, ‘KIF2C’, ‘BUB1’]*
Mitotic Metaphase and Anaphase	1.20 × 10^−11^	*[‘UBE2I’, ‘UBE2C’, ‘CDCA5’, ‘PLK1’, ‘CDCA8’, ‘CENPA’, ‘PSMA7’, ‘CDC20’, ‘CENPE’, ‘DSN1’, ‘KIF18A’, ‘TUBA3C’, ‘PSMA1’, ‘PTTG1’, ‘ESPL1’, ‘NUP85’, ‘CENPI’, ‘TUBB3’, ‘CDC26’, ‘NUF2’, ‘KIF2C’, ‘BUB1’]*
Mitotic Prometaphase	4.51 × 10^−10^	*[‘ODF2’, ‘CDCA5’, ‘PLK1’, ‘HAUS4’, ‘CDCA8’, ‘SMC4’, ‘CENPA’, ‘CDC20’, ‘CENPE’, ‘DSN1’, ‘KIF18A’, ‘TUBA3C’, ‘NUP85’, ‘CENPI’, ‘TUBB3’, ‘CEP70’, ‘NUF2’, ‘KIF2C’, ‘NEK2’, ‘BUB1’]*
Resolution of Sister Chromatid Cohesion	0.0001	*[‘CDCA5’, ‘PLK1’, ‘CDCA8’, ‘CENPA’, ‘CDC20’, ‘CENPE’, ‘DSN1’, ‘KIF18A’, ‘TUBA3C’, ‘TUBB3’, ‘NUP85’, ‘CENPI’, ‘NUF2’, ‘KIF2C’, ‘BUB1’]*
Mitotic Spindle Checkpoint	0.0001	*[‘UBE2C’, ‘PLK1’, ‘CDCA8’, ‘CENPA’, ‘CDC20’, ‘CENPE’, ‘DSN1’, ‘KIF18A’, ‘NUP85’, ‘CENPI’, ‘CDC26’, ‘NUF2’, ‘KIF2C’, ‘BUB1’]*
Cell Cycle Checkpoints	0.0001	*[‘CDKN2A’, ‘UBE2C’, ‘PLK1’, ‘CDCA8’, ‘CDC25C’, ‘PKMYT1’, ‘CENPA’, ‘PSMA7’, ‘CDC20’, ‘CENPE’, ‘DSN1’, ‘KIF18A’, ‘WEE1’, ‘PSMA1’, ‘CCNE2’, ‘NUP85’, ‘CENPI’, ‘CDC26’, ‘RPA3’, ‘NUF2’, ‘KIF2C’, ‘BUB1’]*
EML4 and NUDC in Mitotic Spindle Formation	0.0001	*[‘PLK1’, ‘CDCA8’, ‘CENPA’, ‘CDC20’, ‘CENPE’, ‘DSN1’, ‘KIF18A’, ‘TUBA3C’, ‘TUBB3’, ‘NUP85’, ‘CENPI’, ‘NUF2’, ‘KIF2C’, ‘BUB1’]*
Amplification of Signal From Unattached Kinetochores via a MAD2 Inhibitory Signal	0.0004	*[‘CDC20’, ‘CENPE’, ‘DSN1’, ‘KIF18A’, ‘NUP85’, ‘CENPI’, ‘NUF2’, ‘PLK1’, ‘CDCA8’, ‘KIF2C’, ‘CENPA’, ‘BUB1’]*
Amplification of Signal From the Kinetochores	0.0004	*[‘CDC20’, ‘CENPE’, ‘DSN1’, ‘KIF18A’, ‘NUP85’, ‘CENPI’, ‘NUF2’, ‘PLK1’, ‘CDCA8’, ‘KIF2C’, ‘CENPA’, ‘BUB1’]*
RHO GTPases Activate Formins	0.0010	*[‘PLK1’, ‘CDCA8’, ‘CENPA’, ‘CDC20’, ‘CENPE’, ‘DSN1’, ‘KIF18A’, ‘TUBA3C’, ‘TUBB3’, ‘NUP85’, ‘CENPI’, ‘NUF2’, ‘KIF2C’, ‘BUB1’]*
Polo-like Kinase Mediated Events	0.0021	*[‘WEE1’, ‘PLK1’, ‘MYBL2’, ‘CDC25C’, ‘PKMYT1’]*
Activation of APC C and APC C Cdc20 Mediated Degradation of Mitotic Proteins	0.0124	*[‘CDC20’, ‘PTTG1’, ‘PSMA1’, ‘UBE2C’, ‘CDC26’, ‘PLK1’, ‘NEK2’, ‘PSMA7’]*
Mitotic G1 Phase and G1 S Transition	0.0134	*[‘POLA2’, ‘CDKN2B’, ‘WEE1’, ‘RRM2’, ‘CCNE2’, ‘PSMA1’, ‘CDKN2A’, ‘RPA3’, ‘GMNN’, ‘MYBL2’, ‘PSMA7’, ‘CKS1B’]*
G2 M Transition	0.0156	*[‘ODF2’, ‘PLK1’, ‘HAUS4’, ‘CDC25C’, ‘PKMYT1’, ‘PSMA7’, ‘TPX2’, ‘WEE1’, ‘TUBA3C’, ‘PSMA1’, ‘TUBB3’, ‘CEP70’, ‘NEK2’, ‘MYBL2’]*
Mitotic G2-G2 M Phases	0.0167	*[‘ODF2’, ‘PLK1’, ‘HAUS4’, ‘CDC25C’, ‘PKMYT1’, ‘PSMA7’, ‘TPX2’, ‘WEE1’, ‘TUBA3C’, ‘PSMA1’, ‘TUBB3’, ‘CEP70’, ‘NEK2’, ‘MYBL2’]*
APC C-mediated Degradation of Cell Cycle Proteins	0.0281	*[‘CDC20’, ‘PTTG1’, ‘PSMA1’, ‘UBE2C’, ‘CDC26’, ‘PLK1’, ‘NEK2’, ‘PSMA7’]*
Regulation of Mitotic Cell Cycle	0.0281	*[‘CDC20’, ‘PTTG1’, ‘PSMA1’, ‘UBE2C’, ‘CDC26’, ‘PLK1’, ‘NEK2’, ‘PSMA7’]*
RHO GTPase Effectors	0.0281	*[‘PLK1’, ‘CDCA8’, ‘NOXO1’, ‘CDC25C’, ‘IQGAP3’, ‘CENPA’, ‘CDC20’, ‘CENPE’, ‘DSN1’, ‘KIF18A’, ‘TUBA3C’, ‘NUP85’, ‘CENPI’, ‘TUBB3’, ‘PRC1’, ‘NUF2’, ‘KIF2C’, ‘BUB1’]*
APC C Cdh1 Mediated Degradation of Cdc20 and Other APC C Cdh1 Targets in Late Mitosis Early G1	0.0361	*[‘CDC20’, ‘PTTG1’, ‘PSMA1’, ‘UBE2C’, ‘CDC26’, ‘PLK1’, ‘PSMA7’]*

**Table 3:** Performance of machine learning-based models.

Model	Dataset	AUC	Accuracy	Sensitivity	Specificity
Decision Tree	TCGA	0.8421	0.9018	0.8856	0.9129
ANN	TCGA	0.9032	0.9431	0.9394	0.9468
RF	TCGA	0.9319	0.9624	0.9576	0.9662
SVC	TCGA	0.9124	0.9516	0.9451	0.9568
AdaBoost	TCGA	0.8917	0.9348	0.9289	0.9396
GBM	TCGA	0.9221	0.9578	0.9529	0.9617
Hybrid GBM+RF	TCGA	0.9526	0.9749	0.9781	0.9692

**Table 4:** Model-specific selected differentially expressed genes identified by machine learning algorithms.

Decision Tree	ANN	RF	GBM	SVC	AdaBoost	Hybrid GBM+RF
*RSAD2*	*KIF23*	*XAF1*	*XAF1*	*APP*	*IFIH1*	*XAF1*
*DLGAP5*	*UBE2D2*	*RPA3*	*IFIH1*	*SNAP23*	*APP*	*APP*
*APP*	*CDKN3*	*APP*	*APP*	*XAF1*	*XAF1*	*RPA3*
*KIF2C*	*RPA3*	*RSAD2*	*RPA3*	*CD80*	*KIF2C*	*IFIH1*
*UBE2C*	*MELK*	*UBE2D2*	*UBE2D2*	*RPA3*	*RPA3*	*UBE2D2*
*XAF1*	*CXCL10*	*PLK1*	*KIF2C*	*CXCL10*	*UBE2D2*	*RSAD2*
*PLK1*	*FCGR3A*	*KIF2C*	*SNAP23*	*KIF23*	*CXCL10*	*KIF2C*
*RRM2*	*RACGAP1*	*MX1*	*PLK1*	*UBE2D2*	*STAT1*	*PLK1*
*MX1*	*SNAP23*	*FCGR3A*	*RSAD2*	*FCGR3A*	*CDKN3*	*SNAP23*
*FCGR3A*	*PLK1*	*SNAP23*	*DDX58*	*IFIH1*	*NUF2*	*DDX58*
*RPA3*	*CD80*	*DDX58*	*DLGAP5*	*TPX2*	*MX1*	*MX1*
*STAT1*	*MX1*	*UBE2C*	*CDKN3*	*STAT1*	*PLK1*	*CDKN3*
*CDC20*	*UBE2C*	*CDKN3*	*STAT1*	*BUB1*	*FCGR3A*	*UBE2C*
*RACGAP1*	*BUB1*	*IFIH1*	*CD80*	*KIF2C*	*SNAP23*	*DLGAP5*
*IFNG*	*APP*	*CDC20*	*UBE2C*	*MELK*	*UBE2C*	*FCGR3A*
*CXCL10*	*RSAD2*	*CXCL10*	*MX1*	*UBE2C*	*IFNG*	*STAT1*
*SNAP23*	*NUF2*	*STAT1*	*BUB1*	*RSAD2*	*CD80*	*CD80*
*KIF20A*	*XAF1*	*KIF23*	*KIF23*	*DLGAP5*	*MELK*	*KIF23*
*UBE2D2*	*TPX2*	*CD80*	*FCGR3A*	*DDX58*	*KIF20A*	*BUB1*
*BUB1*	*KIF2C*	*DLGAP5*	*RACGAP1*	*KIF20A*	*TPX2*	*RACGAP1*

**Table 5:** Performance Metrics of Classification Models on GSE14206 Validation Dataset.

Model	AUC	Accuracy	Sensitivity	Specificity
Decision Tree	0.8520	0.8667	0.7895	0.8837
AdaBoost	0.8945	0.9000	0.8421	0.9128
ANN	0.9085	0.9048	0.8421	0.9186
SVC	0.9012	0.9048	0.8421	0.9186
GBM	0.9150	0.9048	0.8947	0.9070
RF	0.9124	0.9143	0.8947	0.9186
Hybrid GBM+RF	0.9156	0.9153	0.8947	0.9186

**Table 6:** Model-specific selected genes identified in the GSE14206 validation dataset.

Decision Tree	ANN	RF	GBM	SVC	AdaBoost	Hybrid GBM+RF
*RSAD2*	*KIF23*	*XAF1*	*XAF1*	*APP*	*IFIH1*	*XAF1*
*DLGAP5*	*UBE2D2*	*RPA3*	*IFIH1*	*SNAP23*	*APP*	*APP*
*APP*	*CDKN3*	*APP*	*APP*	*XAF1*	*XAF1*	*RPA3*
*KIF2C*	*RPA3*	*RSAD2*	*RPA3*	*CD80*	*KIF2C*	*IFIH1*
*UBE2C*	*MELK*	*UBE2D2*	*UBE2D2*	*RPA3*	*RPA3*	*UBE2D2*
*XAF1*	*CXCL10*	*PLK1*	*KIF2C*	*CXCL10*	*UBE2D2*	*RSAD2*
*PLK1*	*FCGR3A*	*KIF2C*	*SNAP23*	*KIF23*	*CXCL10*	*KIF2C*
*RRM2*	*RACGAP1*	*MX1*	*PLK1*	*UBE2D2*	*STAT1*	*PLK1*
*MX1*	*SNAP23*	*FCGR3A*	*RSAD2*	*FCGR3A*	*CDKN3*	*SNAP23*
*FCGR3A*	*PLK1*	*SNAP23*	*DDX58*	*IFIH1*	*NUF2*	*DDX58*
*RPA3*	*CD80*	*DDX58*	*DLGAP5*	*TPX2*	*MX1*	*MX1*
*STAT1*	*MX1*	*UBE2C*	*CDKN3*	*STAT1*	*PLK1*	*CDKN3*
*CDC20*	*UBE2C*	*CDKN3*	*STAT1*	*BUB1*	*FCGR3A*	*UBE2C*
*RACGAP1*	*BUB1*	*IFIH1*	*CD80*	*KIF2C*	*SNAP23*	*DLGAP5*
*IFNG*	*APP*	*CDC20*	*UBE2C*	*MELK*	*UBE2C*	*FCGR3A*
*CXCL10*	*RSAD2*	*CXCL10*	*MX1*	*UBE2C*	*IFNG*	*STAT1*
*SNAP23*	*NUF2*	*STAT1*	*BUB1*	*RSAD2*	*CD80*	*CD80*
*KIF20A*	*XAF1*	*KIF23*	*KIF23*	*DLGAP5*	*MELK*	*KIF23*
*UBE2D2*	*TPX2*	*CD80*	*FCGR3A*	*DDX58*	*KIF20A*	*BUB1*
*BUB1*	*KIF2C*	*DLGAP5*	*RACGAP1*	*KIF20A*	*TPX2*	*RACGAP1*

**Table 7:** A specimen of the z-scores data of TCGA.

Hugo_Symbol	TCGA-2A-A8VL	TCGA-2A-A8VO	TCGA-2A-A8VT	TCGA-2A-A8VV
*UBE2Q2P2*	0.2808	−0.0291	2.0529	0.2002
*HMGB1P1*	−0.6698	−1.5009	−0.1843	0.2431
*RNU12-2P*	−1.8207	−1.8207	0.6468	−1.8207
*SSX9P*	−2.3983	−2.3983	−2.3983	−2.3983

**Table 8:** Hyperparameter search space and final selected parameters used in the nested cross-validation framework.

Model	Hyperparameter	Search Space	Inner-Loop Criterion	Final Selected Value
GBM	n_estimators	100, 200, 300, 500	AUC	300
GBM	learning_rate	0.01, 0.05, 0.10	AUC	0.05
GBM	max_depth	2, 3, 4, 5	AUC	3
GBM	subsample	0.7, 0.8, 1.0	AUC	0.8
RF	n_estimators	100, 300, 500, 1000	AUC	500
RF	max_depth	None, 5, 10, 20	AUC	10
RF	max_features	sqrt, log2	AUC	sqrt
RF	min_samples_split	2, 5, 10	AUC	2
Hybrid GBM+RF	GBM component	Best inner-loop GBM configuration	AUC	n_estimators = 300; learning_rate = 0.05; max_depth = 3; subsample = 0.8
Hybrid GBM+RF	RF component	Best inner-loop RF configuration	AUC	n_estimators = 500; max_depth = 10; max_features = sqrt; min_samples_split = 2

## Data Availability

The datasets analyzed in this study are publicly available. TCGA Prostate Adenocarcinoma (PanCancer Atlas) data are available through cBioPortal, and the external validation dataset GSE14206 is available through the Gene Expression Omnibus (GEO).

## Abbreviations

The following abbreviations are used in this manuscript:

Abbreviation	Definition
AI	Artificial intelligence
ANN	Artificial neural network
AUC	Area under the curve
CI	Confidence interval
DEG	Differentially expressed gene
FDR	False discovery rate
GBM	Gradient boosting machine
GCFS	Graph-convolutional feature selection
GEO	Gene Expression Omnibus
GO	Gene Ontology
ISG	Interferon-stimulated gene
KEGG	Kyoto Encyclopedia of Genes and Genomes
ML	Machine learning
PCa	Prostate cancer
PPI	Protein–protein interaction
PRAD	Prostate adenocarcinoma
RF	Random forest
RNA-seq	RNA sequencing
ROC	Receiver operating characteristic
RSEM	RNA-Seq by Expectation Maximization
SVC	Support vector classifier
TCGA	The Cancer Genome Atlas
